# Fine structural aspects on the web glue production in the golden orb-web spider *Trichonephila clavata*

**DOI:** 10.1080/19768354.2023.2168753

**Published:** 2023-01-30

**Authors:** Yan Sun, Seung-Min Lee, Bon-Jin Ku, Myung-Jin Moon

**Affiliations:** Department of Biological Sciences, Dankook University, Cheonan, Korea

**Keywords:** Spider, silk, fine structure, web glue, *Trichonephila clavata*

## Abstract

The water-soluble glue substance of the capture threads in *Trichonephila clavata* is solely produced from two pairs of aggregate silk glands. During the web glue production, secretory vesicles were synthesized via the extensive rough endoplasmic reticulum of epithelial cells. Unlike the clearly described fibrous web production in spiders, the process of aqueous web glue production appears to involve either a condensing or a packaging step by the Golgi complex. In particular, the fine structure of secretory vesicles varies from cell to cell and may represent the secretory cycle. The electron-dense multivesicular bodies were clearly visible as discrete droplets, and the mature secretory product in the glandular epithelium appeared as a spherical vacuole grown by fusion with surrounding small vesicles. Our fine structural observation reveals that the secretion occurs when the release of secreted material involves the loss of part of the cytoplasm. The bleb along the luminal surface of the secretory cells and membrane-bound extracellular vesicles which pinched off from the cell suggests that the secretory product is released by the mechanism of apocrine secretion.

## Introduction

The viscous prey capture threads of the web-building spiders are produced from the substances of the aggregate silk gland (ASG) and flagelliform silk gland (FSG) (Peters and Kovoor 1991; Moon and Kim 2005). Previous research has shown that the capture thread of an orb-web spider comprises only one type of silk fiber which is originated from the FSG of spiders (Römer and Scheibel 2008). In addition, it has been widely known that spider’s web glue is basically a viscous solution produced from the ASGs and coats the spiral threads of spider web for prey capture (Choresh et al. 2009).

Subsequent studies have confirmed that the main component of the glue contained within microscopic nodules is made of a glycoprotein (Vollrath and Tillinghast 1991; Tillinghast et al. 1993). The supporting fibers of sticky capture threads are wrapped with a complex aqueous solution that integrate into droplets that obtain their adhesiveness from glycoprotein within nodules (Vollrath et al. 1990; Townley et al. 1991; Vollrath 1992). Hawthorn and Opell (2003) also suggested that the content of this solution could create hygroscopic forces, which could contribute to the stickiness of threads. As a result of chemical analysis of this aqueous gluey substances, the concentration of related water-soluble organic compounds such as free amino acids, small peptides, and neurotransmitters was relatively high, while the concentrations of various inorganic salts and glycoproteins were relatively low (Vollrath and Tillinghast 1991; Townley et al. 2006; Römer and Scheibel 2008).

Since the gluey substances in orb-web spiders are reported as one of the strongest biological glue (Vollrath et al. 1990; Tillinghast et al. 1993), spider web glues that coat the sticky spirals of the capture threads have attracted researchers to analyze their unique biochemical characteristics and industrial potential to create new biomaterials (Choresh et al. 2009). This biological material that provides adhesion and thread viscosity is originated from regularly spaced droplets, whose size and spacing are determined by the diameters of the axial fibers, amount of depositions, and viscosity of the aqueous solution (Vollrath and Tillinghast 1991; Opell and Hendricks 2010).

In addition, the granular size of the glycoprotein produced from the ASG can affect not only the droplet size (Vollrath and Tillinghast 1991), but also the hydrophilic compounds and atmospheric moisture that prevent the droplets from drying out (Townley et al. 1991). With the exception of some brief studies (Moon and Kim 2005; Park and Moon 2014; Moon 2018), fine structural visualization of the ASG has been nearly ignored. So far, information on the cellular process of web glue production in the spider has been limited. Therefore, we describe here the fine structural frameworks of the ASG cells that contribute to the production of massive amount of gluey materials in the spider, *Trichonephila clavata* with aid of high-resolution transmission electron microscope for biological sciences (Bio-TEM).

## Materials and methods

The golden orb-web spider, *Trichonephila clavata* (Araneae: Nephilidae), were collected in a local area near the cheonan campus of Dankook University, Cheonan, Korea. *Trichonephila* is a genus of orb-weaver spiders belongs to the Araneidae family as a subgenus of *Nephila*. Since *Trichonephila* was elevated to the level of genus in 2019 by Kuntner et al., this species was moved from the genus *Nephila* to *Trichonephila*.

All spiders were reared in an ambient environment with natural light in wooden frame enclosures (height × length × width = 50 × 50 × 10 cm) with front and back glass windows, and fed insect larvae and water.

For transmission electron microscopic examination, adult spiders were anesthetized with carbon dioxide and dissected under a stereoscopic microscope with aid of spider Ringer's solution that contains 160 mM NaCl, 7.5 mM KCl, 20 mM glucose, 4 mM CaCl_2_, 4 mM NaHCO_3_, 1 mM MgCl_2_, pH 7.4 (Moon and Tillinghast 2020). After dissection, specimens were prefixed using a 2% paraformaldehyde and 2.5% glutaraldehyde mixture solution buffered with 0.1 M phosphate buffer at pH 7.4. The specimens were then postfixed with 1% OsO_4_ in the same buffer and rinsed repeatedly with 0.1 M phosphate buffer (Sun et al. 2020). Following fixation, dehydration procedure was performed in ascending series of ethanol concentrations and embedded in Poly/Bed 812-Araldite Embedding Media (Polysciences Inc., Warrington, PA, USA) via propylene oxide as an intermediary for infiltration (Kim and Moon 2018).

Semi-thin sections with the thickness of 0.5–1.0 µm for light microscopic observation were acquired with a LKB Ultratome V (LKB, Stockholm, Sweden), then cover sections with 1% toluidine blue staining solution and heat on a hot plate at 60°C. Images were captured digitally on a Zeiss Axiophot microscope (Carl Zeiss, Jena, Germany) using a Motic digital camera (Motic Instruments Inc., Richmond, BC, Canada) with on-chip integration.

Ultrathin sections for TEM imaging were prepared using a diamond knife (Ultra 45° Diatome, Hartfield, PA, USA). The specimens were stained with alcoholic uranyl acetate and lead citrate. The sections were then examined precisely with a transmission electron microscope supplied by JEOL (JEM 100 CX-II, JEOL Ltd., Tokyo, Japan) at an accelerating voltage of 80 kV.

## Results

**Figure 1.**
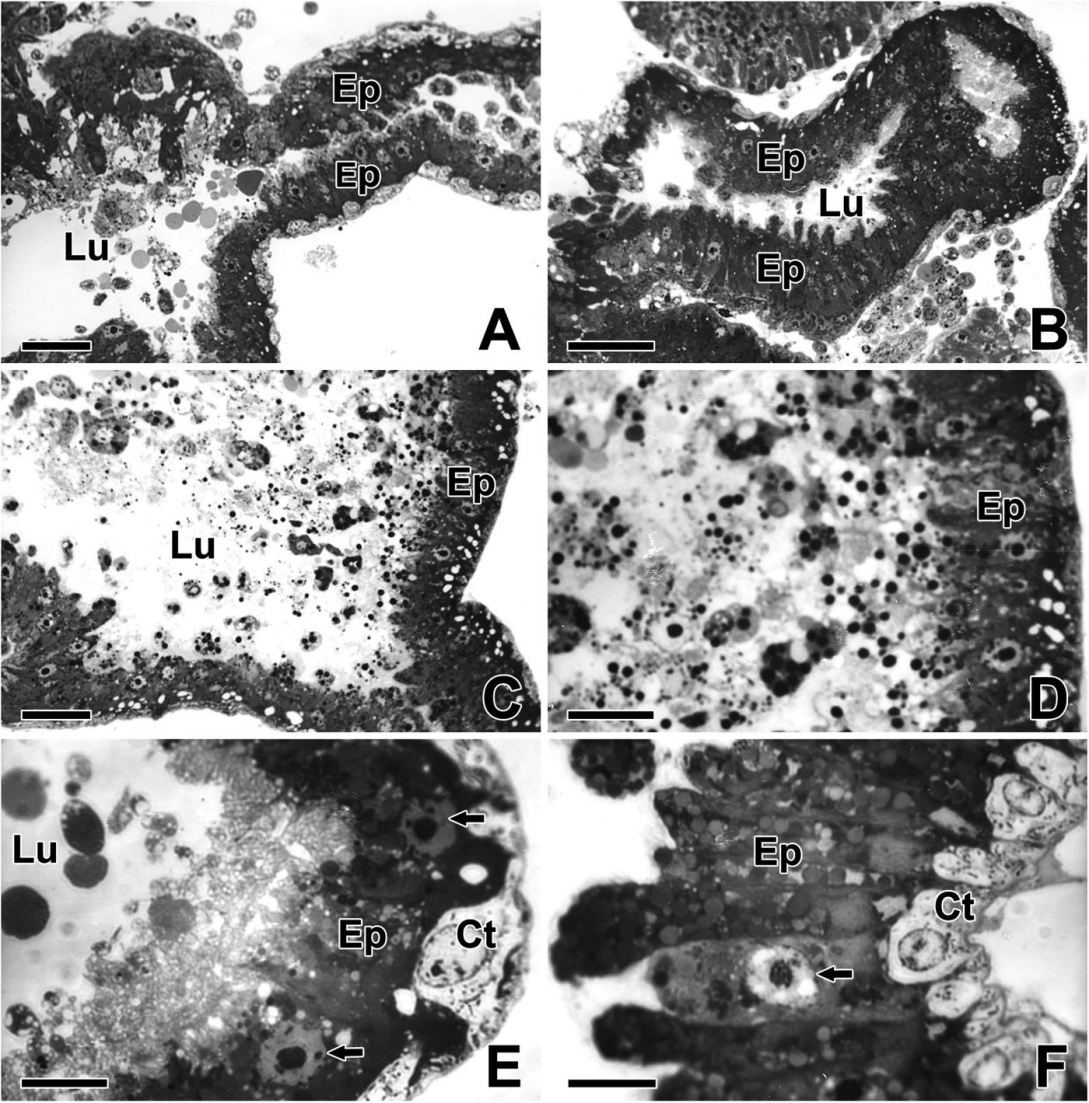
Photo micrographs of the ASG in *T. clavata*. (A, B) The secretory sac of the aggregate gland is widely extended and the glandular epithelium (Ep) of the aggregate gland is basically composed of a single layer of cuboidal cells. Lu: lumen. (C, D) In transverse section, the glands show a wide lumen filled with secretions. The secretory sac is basically consisted of inner epithelial cells and outer connective cells. (E, F) The nuclei of the epithelial cells (arrows) are spherical and occupy more than half the width. Ct: connective cell. Scale bars indicate 250 μm (A–C), 100 μm (D), and 20 μm (E, F), respectively.

The ASG of *T. clavata* is basically a sac. The opening of the gland is connected to an excretory duct leading to the spigot of the posterior spinneret. The secretory sac of the ASG is widely extended across the bordering area of the other opisthosomal tissues (Figure 1(A,B)). Plastic-embedded sections stained with toluidine blue clearly show their convoluted morphology and histologic organization. Histologically, the secretory sac is consisted of inner epithelial cells and outer connective cells, and they are surrounded by a thin basal lamina (Figure 1(C,D)). In transverse section, the ASGs are consisted of a multi-lobed secretory region surrounded by a single layer of the cuboidal epithelium and a widely dilated lumen that stores the secretory products. The nucleus of epithelial cell is spherical in shape and it occupies about half of the volume of the cell (Figure 1(E,F)).

**Figure 2.**
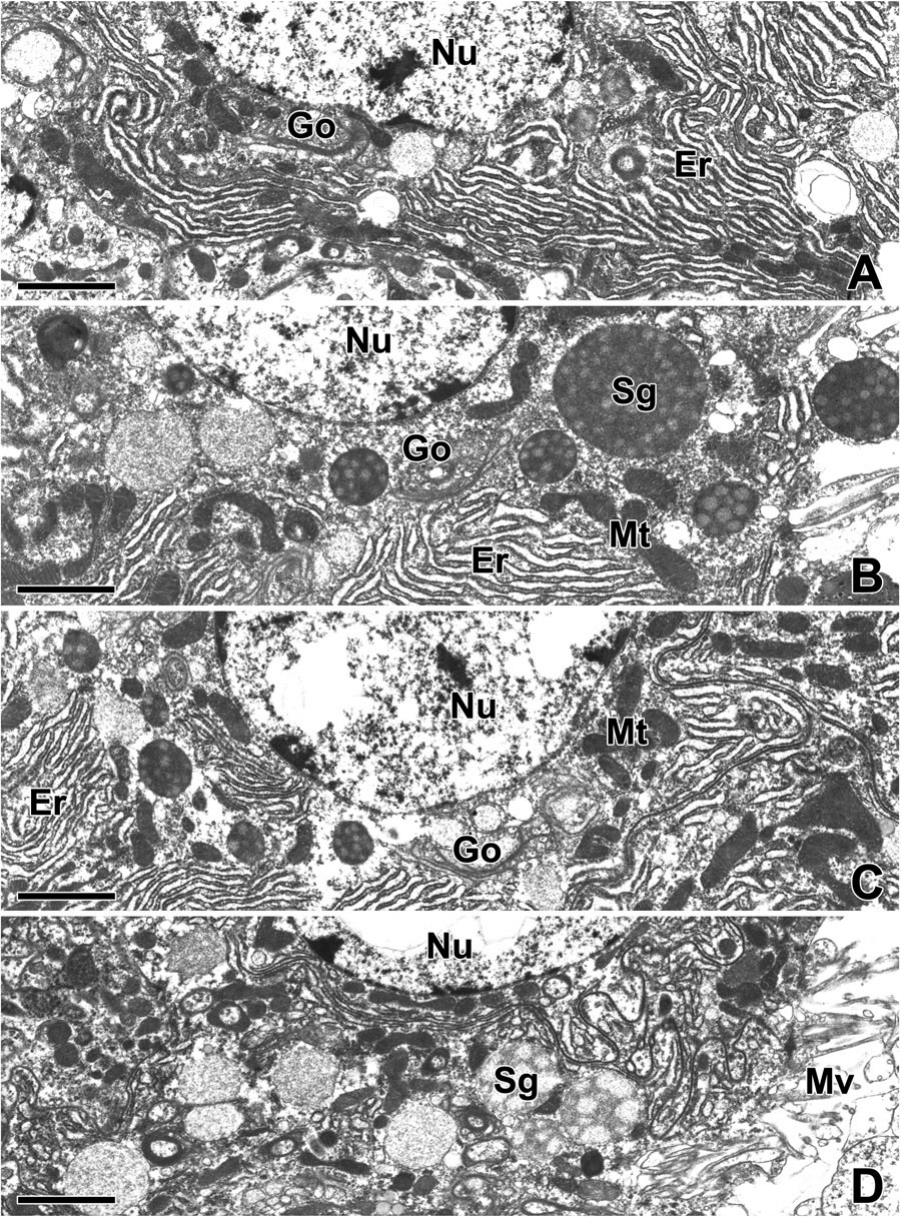
Transmission electron micrographs of the aggregate gland in *T. clavata*. (A) Secretory cells at the basal portion are filled with abundant cisternae of rough endoplasmic reticulum (Er) and their membrane is generally digitated. Golgi apparatuses (Go) appeared in the cytoplasm near the nucleus (Nu). (B) The cell at the middle portion is also occupied by a large amount of rER, mitochondria (Mt) and secretory granules (Sg) with multivesicular appearance. (C) Secretory droplets at apical pole are moved towards the apical border of the cell. Each droplet contains a material of low electron opacity with a multivesicular appearance. (D) Along the apical border of the cell, secretory granules and numerous microvilli (Mv) are projecting into the lumen.

Since the secretory cells of ASG are filled with cisternae of rough endoplasmic reticulum (rER), the limiting membranes between adjacent cells cannot be easily distinguished. The plasma membrane at the base of cells is generally digitated. Deep and complex infoldings are usually found near junctions between adjacent cells (Figure 2(A)). The central part of the cell is occupied by a large nucleus, mitochondria, Golgi complexes, and large amounts of rER. The rER is uniformly distributed throughout the cytoplasm but is missing in areas near the apical and basal borders. The Golgi complex is also sparsely scattered near the apical cytoplasm of the cells (Figure 2(B)).

The cytoplasm below the apical surface is filled with a variety sizes of secretory droplets ranging from 1.0 to 3.5 µm in diameter. These droplets gradually migrate into the apical cytoplasm and are finally extruded towards the lumen of the ASG. Each droplet contains a low electron opacity material with a multivesicular appearance (Figure 2(C)). There are numerous microvilli that protrude into the lumen of the gland along the apical border of the cell. The finger-like projections of the microvilli are bounded by the continuation of cell membranes. Secretory droplets are frequently found migrating into the gland lumen across the apical border of the cell (Figure 2(D)).

**Figure 3.**
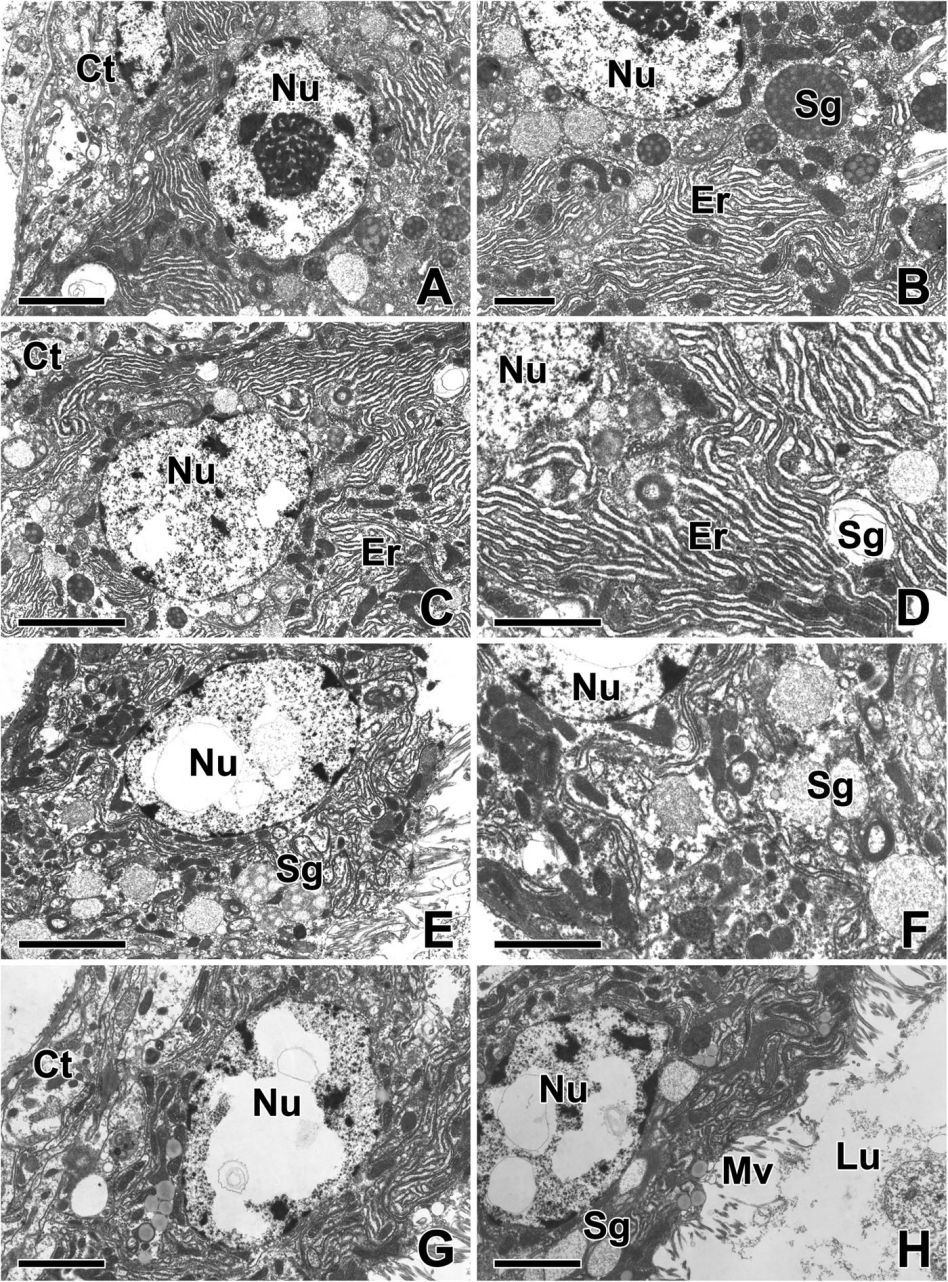
Transmission electron micrographs of the aggregate gland in *T. clavata*. (A) Each epithelial cell shows a large nucleus (Nu) with fine granular chromatin and a prominent nucleolus. Ct: connective cell. (B) An extensive rER (Er) and numerous secretory granules (Sg) with electron-dense multivesicular bodies are observed in the cytoplasm. (C, D) Well-oriented rER cisternae are scattered from base to apex of the cells, particularly near the nucleus. (E, F) Secretory granules are clearly visible with appearance of multivesicular vesicles in the cytoplasm. (G, H) The luminal surface are modified into numerous microvilli (Mv) and the final electron-dense granules are attached to the microvilli. Scale bars indicate 5 μm (A, C–H) and 2 μm (B).

Because the glandular epithelial cells of the ASG actively synthesize and release specific gluey substances to the capture thread, each individual cell has an active cytoplasm accompanied by a spherical nucleus with condensed chromatin and a distinct contrasted nucleolus (Figure 3(A)). In particular, the entire remaining space in the cytoplasm is mostly occupied by the extended rER. Numerous secretory vesicles containing high electron density presumably precursors of gluey substances can be seen in the apical cytoplasm of this epithelium (Figure 3(B)).

Our TEM results clearly show that extensive rER synthesize numerous secretory vesicles in the cytoplasm and subsequent vesicular fusion of multiple vesicles can create intensive production of secretory vacuoles (Figure 3(C,D)). One of the prominent features of the cell nucleus at this stage is electron-lucent chromatin and an enlarged nucleolus. Additionally, exocytotic release of individual droplets can be seen in this stage of glandular epithelial cells. Since regularly oriented rER cisternae accumulate in abundance from basal to apex of the cells, it can be assumed that the major component of the gluey substance contained in the cytoplasm of ASG is derived from the rER of glandular epithelial cells (Figure 3(E,F)).

Compared to the early stage of cells, the epithelium at the later stages of extrusion exhibits thin columnar cells with indistinct cell membranes. There cells contain multiple droplets of secretion, resulting in a much thinner staining of the area (Figure 3(G)). These secretory droplets migrate towards the apical border and are finally released towards luminal cavity as a pinch-off portion of the cell during the active process of web glue production. The plasma membrane on the apical pole of the cell is transformed into small finger-like projections of microvilli, and the final secretion of the cytoplasm is anchored to the apical membrane of the microvilli (Figure 3(H)).

**Figure 4.**
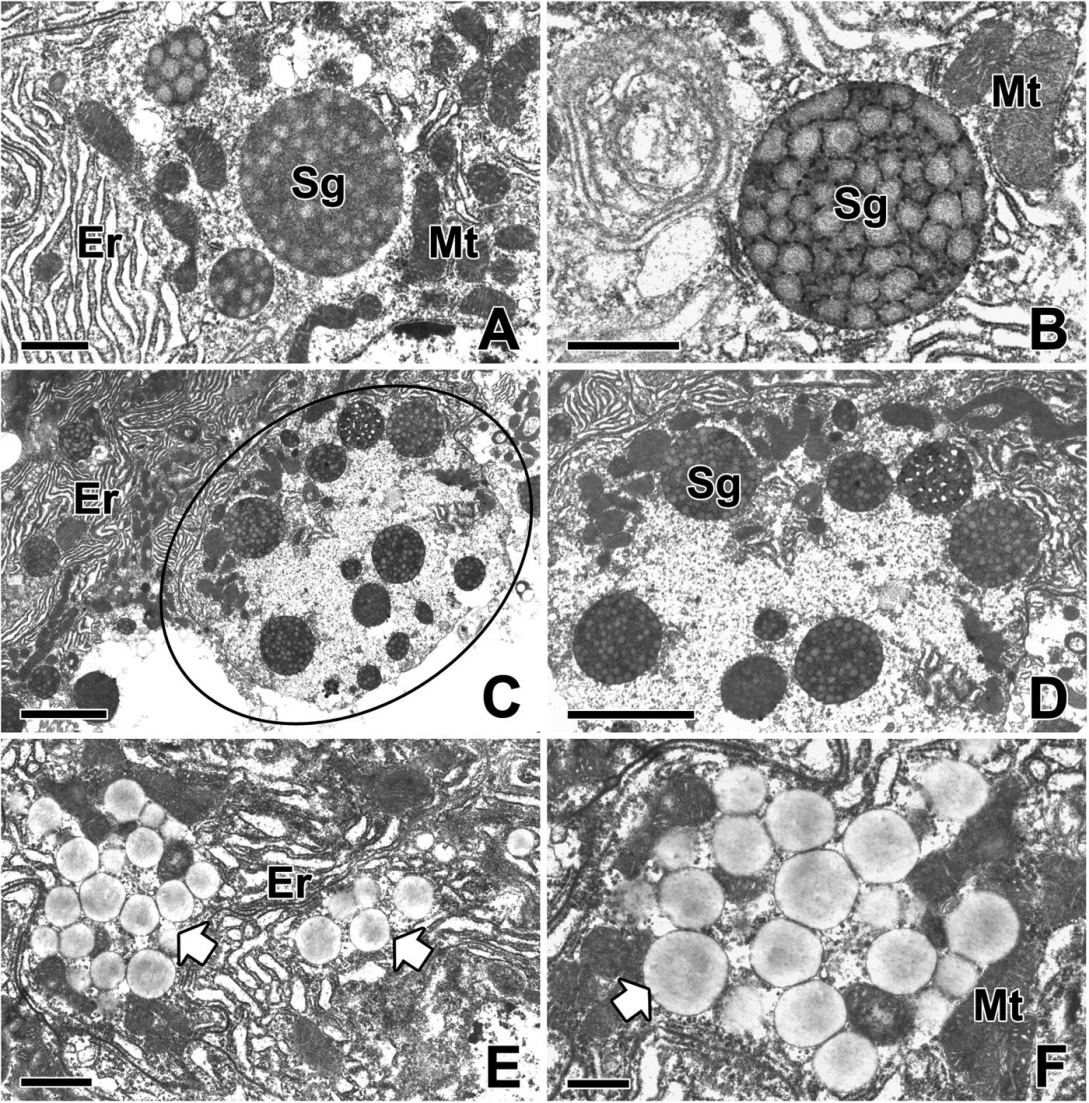
Transmission electron micrographs of the secretory products of the aggregate gland in *T. clavata*. (A, B) The mature secretory granules (Sg) contain electron-dense multivesicular bodies. Er: rER, Mt: mitochondria. (C) A part of apical cytoplasm of the epithelial cell is pinched off and enters the lumen (black circle). (D) The secretory products are compactly aggregated as a single vacuole. (E, F) Another type of secretory silk products are accumulate in the luminal cytoplasm as a form of electron-lucent spherical granules (white arrows). Scale bars indicate 2 μm (C, D), 1 μm (A, B, E), and 0.5 μm (F), respectively.

The main secretory products of the ASG can be observed as large and round vacuoles approximately less than 2 µm in diameter. Some of these vacuoles exhibit multivesicular properties. They contain multiple inner vesicles enclosed within a single outer membrane. Their electron density is relatively high and they remain close to each other without fusion with others (Figure 4(A)). The structural analysis of secretory products can represent the secretion process because it shows some extent of morphologically pattern following the cell cycle. Therefore, our current TEM investigations reveal the fine structural changes in the secretory material from the initial secretory vesicles to mature secretory vacuoles during web glue production in ASG (Figure 4(B)).

It was first observed through this research that secretory substances produced from the ASG of spider located in the apical cytoplasm are released extracellular space by the mechanism of apocrine secretion. It is obvious that the cell involves budding of the apical surface and loses some part of its cytoplasm during this secretion process. Finally, part of the apical cytoplasm of the secretory cells is released with secretion and then enters the lumen (Figure 4(C,D)). In addition, another type of silk precursor is also observed in the cytoplasm of ASG, where these secretory products are found to accumulate in the luminal cytoplasm of glandular epithelial cells in the form of electron-lucent spherical granules. Apparently, the electron density of such granules is much lighter than that of the vacuoles containing multivesicular bodies (Figure 4(E,F)).

The release pathway of the gluey substance across the cell is perceived from the fine structural modifications of the secreted material. It has been observed that disorganization of the secretion finally occurs when the secretory product is extruded from the cell by a specific secretory process. It is observed that the apices of cells are surrounded by numerous microvilli with short and irregular shapes.

**Figure 5.**
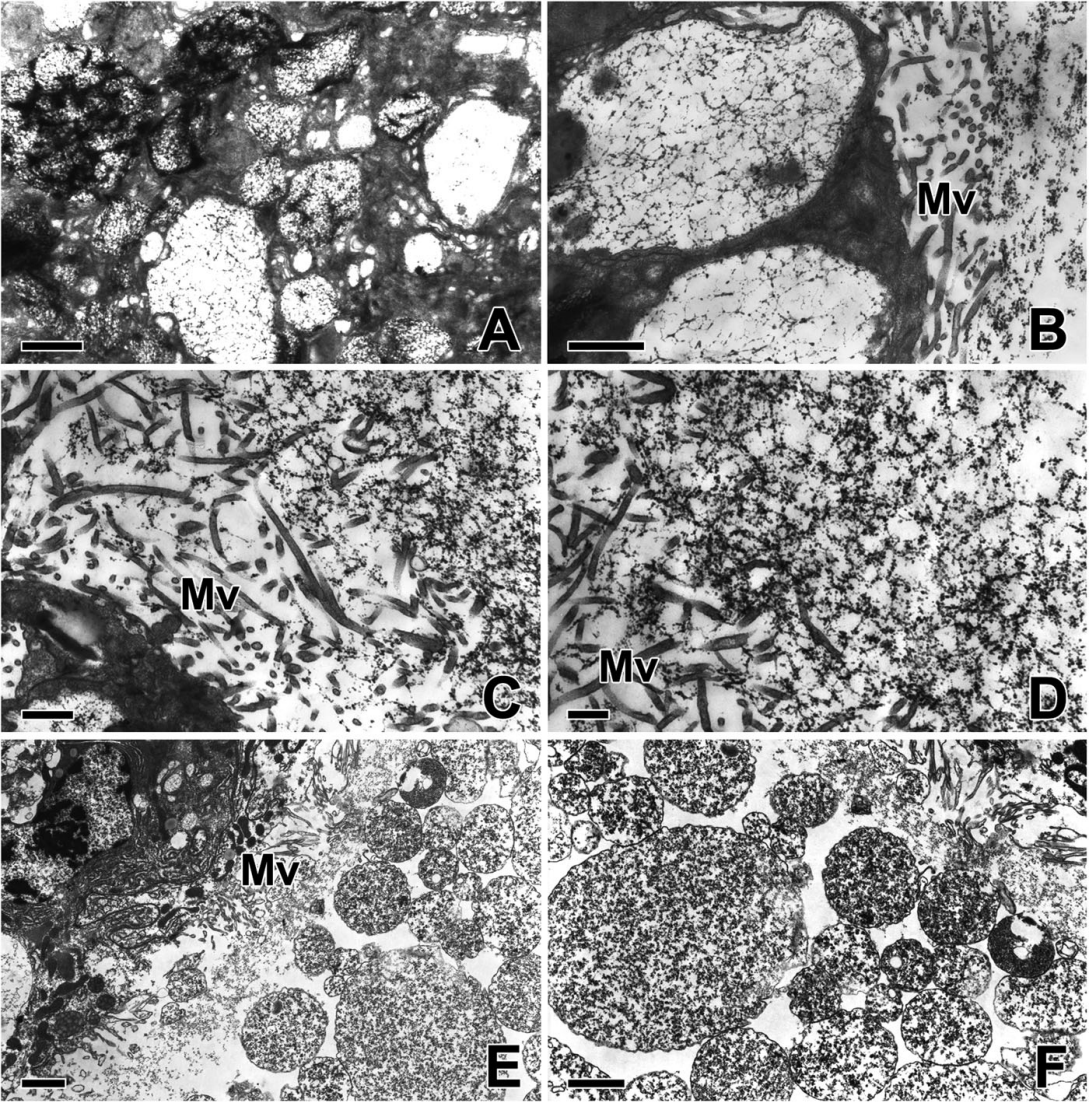
Transmission electron micrographs of the aggregate gland in *T. clavata*. (A, B) When the secretory products are extruded from the cells, the multivesicular vesicles are transformed into secretory vesicles filled with a fine fibrillar substructure. The apices of the glandular cells are fringed with short and irregular microvilli (Mv). (C, D) During the active release of secretory silk products, amorphous electron-dense deposits appear to aggregate with several others. (E, F) The fine granular material is added to the luminal secretory products for the gluey substances. Scale bars indicate 1 μm (A–D) and 2 μm (E, F).

With gradual maturation of secretory silk products, multivesicular bodies are progressively converted to more transparent secretory vesicles filled with fine fibrillar substructure. Thus, mature vesicles are much more electron-lucent than earlier stage vesicles (Figure 5(A,B)).

The total amount of fine fibrous substances gradually increases while the gluey silk is actively released. Frequently, these vesicles appear to accumulate with others to form amorphous deposits with high electron densities (Figure 5(C,D)). These fine granular substances likely migrated across the apical membrane by exocytotic activity, and can be added to the final secretory product as another precursor for gluey substances (Figure 5(E,F)).

**Figure 6.**
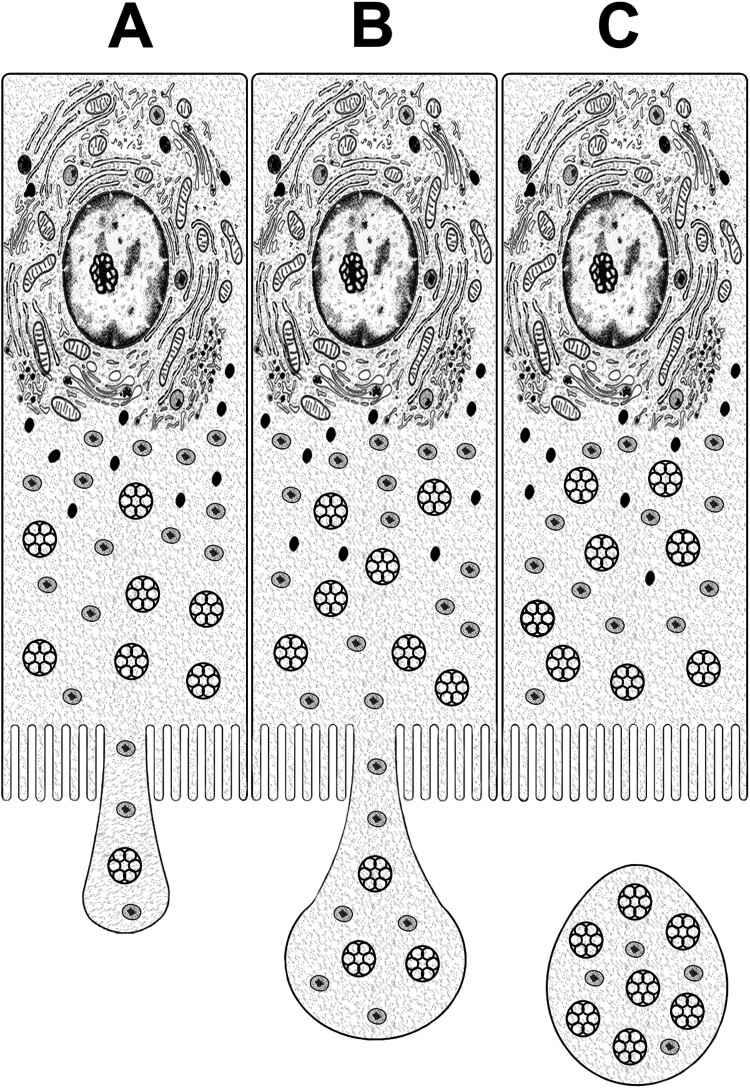
Schematic diagram of the apocrine secretion during the web glue production in the aggregate gland of the orb-web spider, *T. clavata*. The sequence of the process leading to secretion occurs from A to C.

Our fine structural observation clearly shows the secretory process of the glandular epithelum involves the loss of part of the cytoplasm during the web glue production. Moreover, the secretory products accumulated in the secretory vesicles and bleb on the apical cytoplasm of the cells which pinched off from the cell suggest that the web glue material of the aggregate gland is released from the glandular epithelium by the mechanism of apocrine secretion (Figure 6).

## Discussion

Although it is thought that the silk glands of invertebrates produce only physically fibrous silk, in fact liquid silk material is also produced in web-building spiders (Foelix 2010). However, a very limited part of the liquid silk has been investigated to date to understand the effect of the adhesive droplets of capture threads in ecribellate spider. As far as the capture thread is concerned, studies have been largely examined to stickness and extensibility of glue substance (Opell 2002), and the humidity effect of the sticky droplets (Opell and Hendricks 2010; Opell et al. 2011).

Considered one of most effective biological glues, spider silk glue is an aqueous solution produced by the ASGs of orb weaving spiders (Moon and Kim 2005; Choresh et al. 2009). Previous studies have reported that the glue of ecribellate spiders is composed of microscopic nodules, and this glue substance coats the prey-capturing threads of orb-web spiders (Park and Moon 2014; Moon 2018).

Chemical analysis of the sticky and regularly spaced droplets of the capture threads has demonstrated that ASG produces complex aqueous solution of organic and inorganic compounds composed of various types of small proteins (Vollrath et al. 1990; Vollrath and Tillinghast 1991) and high molecular weight glycoproteins (Tillinghast 1981; Townley et al. 1991).

Tillinghast et al. (1993) purified a glycoprotein from *Argiope aurantia* and found a poly disperse linear macromolecule that exhibits structural flexibility. Additionally, the glycoprotein shares some morphological features with mammalian secretory mucins as we also observed in our electron microscopic examination. This is consistent with other reports analyzing spider web glue proteins (Vollrath et al. 1990). Proteins such as mucin are known to have adhesive properties due to their highly glycosylated nature, and their elasticity is known to affect the formation of extended nodules (Choresh et al. 2009).

In *T. clavata*, each ASG showed a multi-lobed secretory region surrounded by a simple layer of the cuboidal cells with large nucleus. These cells are preliminary filled with abundant cisternae of rER, but the Golgi complex also appeared near the apical surface of the cytoplasm. The highly developed rER occupies the whole area of the cytoplasm in the epithelial cells of the ASGs. During the process of web glue production, small vesicles are formed by the rER and these vesicles are packaged into secretory vacuoles and travel through the Golgi complex before releasing secretory substances into lumen. These excessive contents of rER showed morphological signs suggestive of active synthesis of substances within cytoplasm (Nagashima et al. 1991) and mass release of secretions through the luminal space (Locke and Huie 1976).

It is generally known that secretory granules are produced from ribosomes attached to the limiting membranes of the rER in the exocrine cells (Siekevitz and Palade 1960). They are then transported across this membrane, segregated within the cisternae of the rER (Redman et al. 1966), and move to small peripheral vesicles of the Golgi complex (Jamieson and Palade 1967). They are then concentrated in condensed vacuoles in the Golgi complex and finally formed as individual secretory granules (Caro and Palade 1964).

In particular, previous researches have clearly shown that the precursors of dragline silk are produced in a form of ready-to-secretion and do not undergo further concentration process (Bell and Peakall 1969). It has been also reported that the secretory cells of spider silk glands may have developed a unique method to more quickly produce large amount of protein by evolution (Moon et al. 1998). The secretory precursors of the major ampullate glands in the eccribellar spiders including *Nephila* (Moon and Kim 2005), *Argiope* (Moon and Kim 2005), and *Araneus* (Moon and Tillinghast 2020) species showed exactly the similar characteristic of secretion. In addition, Sasaki et al. (1981) studied morphological and biochemical traits of the glandular epithelial cells of the silk gland in the silk moth *Bombyx mori*. They reported that the secretory silk materials were produced as small vesicles through the well-developed rER. In addition, they found that these secretory cells lack the Golgi complex, a cell organelle known to be responsible for modifying and packaging steps of cell secretion.

However, the process of aqueous web glue production in ASGs is different from the fibrous silk production in spiders. Our present study clearly showed that the Golgi complex is also found near the apical surface of cytoplasm, and it is therefore likely that the Golgi complex also plays an important role in the secretory process of web glue production.

Recently, the secretory function of the Golgi complex appears to be well-established experimentally. Therefore, the Golgi complex is known to play a central role in protein synthesis controlling cargo-sorting and trafficking. This is because these processes are functionally important for cell polarity, motility, division, and growth (Park et al. 2021). This is consistent with our fine structural observations of ASGs in *T. clavata*, but their contribution to web glue production seems to be very limited because the Golgi cisternae remain unreactive and the condensed vacuoles in the trans Golgi region lack dense cores.

The apocrine secretion is an alternative extrusion mechanism of membrane-associated proteins (Aumüller et al. 1999) when secretory granules accumulate in the cytoplasm of a cell, a part of the cytoplasm surrounds and pinches off the granules (Farkaš et al. 2014). Although exocytosis is commonly regarded as a basic type of secretion, the apocrine secretory mechanism has not been properly described because the pathways that control the secretory process remain obscure (Gesase and Satoh 2003). The apocrine glands are found primarily in the mammary milk glands of breast and apocrine sweat glands. Secretion from apocrine glands contains are made up of proteins and fatty acids, therefore they are viscid and odorous (Spielman et al. 1998).

The ASG preparation in *T. clavata*, secretory products are finally extruded into the gland lumen across the apical border of the cell after packaging steps of cell secretion. It is obvious that the cell involves the budding of apical surface and loses some of its cytoplasm in this process of secretion. The ASG is characterized by a simple epithelium and widely dilated lumen that stores the secretory product. Secretory granules are gathered at the apical area of the cytoplasm, and they are extruded to the gland lumen. Our electron microscopic observations apparently show that this process is accompanied by the release of large fragments of cellular contents and loss of certain part of the cytoplasm. In particular, the small apical protrusions seen in the cytoplasm suggest that the secretory materials are finally released by the mechanism of apocrine secretion (Kurosumi and Kawabata 1976).

Following the observations in *Araneus sericatus* (Bell and Peakall 1969), Candelas and Lopez (1983) reported their observations on the apocrine secretion in the process of spider silk production. In addition, Farkaš et al. (2014) also reported the apocrine secretion in *Drosophila* salivary glands, recently. It has been known that such apocrine secretion is more damaging than merocrine secretion (Schneider and Paus 2010) but provides the en masse delivery of a protein mixture from polarized epithelial tissues (Farkaš et al. 2014).

Comparisons of the results of this research with other studies on fibrous silk production, strengthen the premise that the aqueous web glue (liquid silk) production process is different from the fibrous silk production of spiders. Evolutionally, the orb-web spiders produce a remarkable variety of webs (Hormiga and Griswold 2014) and contents of the ASG are known to have evolved later than other spider silk proteins as well as other silk glands (Choresh et al. 2009). In addition, it has been previously noted that large multi-lobed ASGs may have been produced by the coalescence of multiple small silk glands (Kovoor 1987; Moon and Kim 2005). These suggest that the secretory process within the ACG is more complex compared to the relatively simple but more massive fibrous silk production.
